# Possibly preventable cardiac arrest in a morbidly obese patient - a comment on the 2015 ERC guidelines

**DOI:** 10.1186/s13049-016-0306-4

**Published:** 2016-10-04

**Authors:** Felix Patricius Hans, Claudia Johanna Maria Hoeren, Phillipp Kellmeyer, Lisa Hohloch, Hans-Jörg Busch, Jörg Bayer

**Affiliations:** 1 University Emergency Department, Medical Center - University of Freiburg, Sir-Hans-A-Krebs-Strasse, 79106 Freiburg, Germany; 2 Department of Anesthesiology and Critical Care, Medical Center - University of Freiburg, Hugstetter Strasse 55, 79106 Freiburg, Germany; 3 Department of Neurology and Neuroscience, Medical Center - University of Freiburg, Breisacher Str. 64, 79106 Freiburg, Germany

**Keywords:** Cardiopulmonary resuscitation, Heart arrest, Obesity, Emergencies, Emergency medical services, Emergency service, Hospital

## Abstract

**Background:**

The incidence of overweight and obesity has been steadily on the rise and has reached epidemic proportions in various countries and this represents a well-known major health problem. Nevertheless, current guidelines for resuscitation do not include special sequences of action in this subset of patients. The aim of this letter is to bring this controversy into focus and to suggest alterations of the known standard cardiopulmonary resuscitation in the obese.

**Case presentation:**

An obese patient weighing 272 kg fell to the floor, afterwards being unable to get up again. Thus, emergency services were called for assistance. There were no signs or symptoms signifying that the person had been harmed in consequence of the fall. Only when brought into a supine position the patient suffered an immediate cardiac arrest. Cardiopulmonary resuscitation was performed but there was no return of a stable spontaneous circulation until the patient was brought into a full lateral position. In spite of immediate emergency care the patient ultimately suffered a lethal hypoxic brain damage.

**Conclusion:**

A full lateral position should be considered in obese patients having a cardiac arrest as it might help to re-establish stable circulatory conditions.

## Letter to the Editor

Increasing numbers of overweight and obese patients require tailored therapeutic approaches to diseases that come along with such alterations of human constitution and metabolism [1]. Especially the acute care and emergency treatment of this subset of patients is challenging and needs to be constantly adapted. Although recently updated, guidelines for cardiopulmonary resuscitation do not mirror those changes in prevalence as no practical instructions are provided to handle morbidly obese patients [2, 3]. We would like to ad a comment to the latest guidelines in the form of a case report, in order to draw attention to this topic and to give a lead in treatment of those patients.

## Case presentation

A 60-year-old patient fell to the floor inside his apartment and alarmed emergency medical services (EMS) consequently. Due to his estimated body mass of >250 kg the patient was unable to get to his feet again by himself. On arrival of the EMS the alert patient lay in a right lateral position and reported no pain, no dyspnea, no chest tightness or any other symptoms suspicious for an acute medical condition or injury. Up to this point, the only disease (apart from obesity) the patient had to deal with was a gonarthrosis. Having experienced such a situation before the patient told the EMS to lift him up again with the help of a “board”. A fire truck was sent to support the EMS team and a backboard was installed to lift the patient. No medical equipment was needed at that time. The moment the patient was moved onto the backboard and brought into a supine position he gasped, his face went cyanotic and he lost consciousness. Without palpable pulse the patient was immediately resuscitated. Cardiopulmonary resuscitation (CPR) was alternately performed by six trained firemen and two paramedics. Medical equipment was installed to support CPR and an emergency physician was called, following the locally implemented protocol. According to the guidelines, the detected asystole was treated by application of epinephrine and the airway was secured by a supraglottic airway device. Return of spontaneous circulation (ROSC) was rapidly achieved consecutively for three times with the ECG showing an atrial fibrillation each time spontaneous circulation returned. Pulses were palpable but the heart rhythm then rapidly degenerated into brady-asystole. The EMS suspected an aorto-caval compression causing the brady-asystole and turned the patient into a full lateral position after the subsequent ROSC - a measure which lead to the patient’s hemodynamic stabilization and allowed transportation to the cardiac arrest center. During the initial clinical workup, a pulmonary embolism was ruled out by CT-scan, laboratory findings revealed a mild myocardial necrosis (interpreted as myocardial infarction type II as there were no regional cardiac wall motion abnormalities). Coronary angiography could not be performed due to the patient’s weight and size. Therapeutic hypothermia was not performed as none of the available systems were considered applicable. During the intensive care stay the patient was transferred in a half-sitting position and remained hemodynamically stable.

The actual patient’s weight proved to be 272 kg. Aortocaval compression could not be reproduced (by ultrasound) under invasive ventilation conditions but was assumed to be the most likely cause of the cardiac arrest. Stimulation of the vagus nerve by the large abdominal mass was considered another potential mechanism for the brady-asystole.

Unfortunately, the patient suffered a hypoxic brain damage to such an extent that he never gained consciousness again and died 5 days after hospital admission.

## Discussion

In this case, a setting - which at first glance seemed to be rather trivial and easy to handle - led to a fatal patient outcome despite instantly initiated high quality CPR by well-trained staff and treatment in a cardiac arrest center. Although never clearly proven, the circumstances mentioned indicate that an aortocaval compression syndrome was the cause of the hear arrest. Morbidly obese patients should therefore be treated with precaution, always bearing in mind the possible physical impact of the patient’s body mass. Based on our experience in the above described CPR setting, especially in PEA/asystole with inefficient chest compressions or rapidly terminating ROSC in the morbidly obese, a tilt maneuver might be considered. With 15° tilts having proven to be ineffective in similar situations we suggest a minimum of 30° to a full lateral tilt as an measure to maintain stable circulatory conditions [4, 5].

## Conclusion

A full lateral position should be considered in obese patients having a cardiac arrest as it might help to re-establish stable circulatory conditions.

## Abbreviations

CPR: Cardiopulmonary resuscitation
CT: Computer tomography
ECG: Electrocardiogram
EMS: Emergency medical services
KG: Kilogram
PEA: Pulseless electric activity
ROSC: Return of spontaneous circulation
